# Efeitos da Heroína nos Parâmetros Eletrocardiográficos

**DOI:** 10.36660/abc.20190296

**Published:** 2020-12-01

**Authors:** Ersin Yildirim, Murat Selcuk, Faysal Saylik, Ferit Onur Mutluer, Ozgur Deniz

**Affiliations:** 1 Umraniye Training and Research Hospital Istanbul Turquia Umraniye Training and Research Hospital, Istanbul - Turquia; 2 Van Egitim ve Arastirma Hastanesi Van Turquia Van Egitim ve Arastirma Hastanesi, Van – Turquia

**Keywords:** Heroína, Dependência de Heroína, Entorpecentes/toxicidade, Arritmias cardíacas/efeitos adversos, Síndrome de QT longo, Edema Pulmonar, Insuficiência Renal, Leucoencefalopatias

## Abstract

**Fundamento:**

Atualmente, o vício em heroína é um problema de saúde preocupante, e as informações sobre os efeitos eletrocardiográficos da heroína são limitadas.

**Objetivos:**

O objetivo do presente estudo é investigar os efeitos da dependência de heroína em parâmetros eletrocardiográficos.

**Métodos:**

Um total de 136 indivíduos, incluindo 66 indivíduos que fumam heroína como grupo de estudo e 70 indivíduos saudáveis sem dependência de drogas como grupo de controle, foram incluídos no estudo. Indivíduos que injetam heroína foram excluídos. A avaliação eletrocardiográfica (ECG) dos usuários de heroína foi realizada e comparada com as do grupo controle. Além disso, os ECGs pré e pós-tratamento do grupo usuário de heroína foram comparados. Um valor de p<0,05 foi aceito como estatisticamente significativo.

**Resultados:**

A frequência cardíaca (77,2±12,8 *versus* 71,4±11,2; p=0,02) foi maior no grupo usuário de heroína em comparação com o grupo controle. Os intervalos QT (341,50±25,80 *versus* 379,11±45,23; p=0,01), QTc (385,12±29,11 *versus* 411,3±51,70; p<0,01) e o intervalo do pico ao fim da onda T (Tpe) (65,41±10,82 *versus* 73,3±10,13; p<0,01) foram significativamente menores no grupo usuário de heroína. Nenhuma diferença foi observada entre os grupos com respeito às razões Tpe/QT e Tpe/QTc. Na análise de subgrupo do grupo usuário de heroína, os intervalos QT (356,81±37,49 *versus* 381,18±40,03; p<0,01) e QTc (382,06±26,41 *versus* 396,06±29,80; p<0,01) foram significativamente mais curtos no período pré-tratamento.

**Conclusão:**

O vício em heroína afeta significativamente os intervalos de tempo QT, QTc e Tpe. Os efeitos de arritmia desses parâmetros já são conhecidos. Os parâmetros eletrocardiográficos desses indivíduos merecem mais atenção. (Arq Bras Cardiol. 2020; 115(6):1135-1141)

## Introdução

A heroína, um depressor do sistema nervoso central (diacetilmorfina), é um opiáceo semissintético. A heroína é um opioide altamente utilizado, e o vício em heroína causa um prejuízo significativo para a sociedade em todo o mundo. A prevalência do uso de heroína aumentou nos últimos anos. A mortalidade entre os usuários de heroína varia entre 1 e 3%, e o método de tratamento mais eficaz para a dependência de heroína é a terapia de reposição de opioides.^1 , 2^ Seu principal efeito adverso é a dificuldade respiratória, que pode levar à morte. Com a perda de tolerância, a overdose de heroína pode ser letal após um período de abstinência. As outras complicações da dependência da heroína, como edema pulmonar,^3^ choque,^4^ lesão miocárdica, insuficiência renal aguda,^5^ rabdomiólise^6^ e leucoencefalopatia,^7^ são descritas na literatura. Além disso, a heroína tem se mostrado eficaz na modulação vagal e regulação autonômica.^8^ No entanto, nosso conhecimento sobre os efeitos cardíacos da dependência de heroína é limitado, o que é um problema de saúde pública importante nesse aspecto. Além disso, há alguns estudos na literatura que mostram a relação entre heroína, toxicidade miocárdica e arritmias.^9 , 10^ Portanto, é essencial compreender as alterações no ECG dependentes de heroína. O objetivo do presente estudo é investigar os efeitos da dependência de heroína sobre os parâmetros eletrocardiográficos.

## Métodos

Após a aprovação do Comitê de Ética, um total de 136 indivíduos foram incluídos no estudo, que incluiu 66 pacientes usuários de heroína via tabagismo e em terapia no Centro de Tratamento e Treinamento para Dependência de Álcool e Drogas, entre 2014 e 2017, como grupo de estudo, além de 70 indivíduos saudáveis sem dependência de drogas que não seja o tabaco como grupo controle. O grupo controle foi selecionado consecutivamente entre os pacientes que visitavam a clínica de cardiologia. A avaliação de ECG daqueles que usam heroína foi realizada e comparada com aqueles do grupo controle. Além disso, foi avaliado o ECG pré e pós-tratamento do grupo usuário de heroína. As características clínicas e demográficas dos pacientes, o estado e a duração da dependência da heroína foram coletados junto aos pacientes e seus prontuários no hospital. Apenas aqueles que fumavam heroína foram incluídos no estudo. Os registros eletrocardiográficos (ECG) dos pacientes foram obtidos com o dispositivo Schiller Cardiovit AT-102 plus, usando a derivação 12 padrão (calibração de 10 mm/mV e taxa de deslizamento de 25 mm/s) na primeira admissão no hospital. Somente os pacientes que recebiam heroína em até 12 horas após os registros de ECG obtidos foram incluídos no estudo. As medições do ECG dos intervalos QT e Tpe foram realizadas manualmente por dois cardiologistas especialistas, usando uma lupa para diminuir os erros de medição. As derivações 2 e V5 foram selecionadas para medir os intervalos QT e Tpe, respectivamente. A média dos três batimentos em cada derivação de ECG foi calculada. O intervalo QT é calculado como o intervalo do início do QRS ao fim da onda T. O intervalo Tpe é definido como o intervalo do pico da onda T ao fim da onda T. Os intervalos QTc foram calculados usando a fórmula de Bazett. O hemograma completo (HC) e os exames bioquímicos foram realizados com Beckman Coulter LH-750 e Beckman Coulter L × 20, respectivamente. Os resultados de cada paciente foram registrados. As avaliações ecocardiográficas de todos os pacientes foram feitas na primeira admissão ao hospital. Todos os participantes foram submetidos à avaliação ecocardiográfica 2D e Doppler (VIVID 3, General Electric, EUA) e a fração de ejeção do ventrículo esquerdo foi calculada usando as regras de Simpson modificadas. Os indivíduos que usaram heroína por via intravenosa, dependentes de álcool, aqueles com doenças das artérias coronárias, insuficiência cardíaca, distúrbios das válvulas cardíacas, arritmias conhecidas, hipertensão, doenças cardíacas congênitas, diabetes, insuficiência hepática ou renal, doença pulmonar obstrutiva crônica, doenças endócrinas, distúrbios metabólicos ou eletrolíticos, infecções agudas ou crônicas ou pacientes que tomaram medicamentos que podem afetar os intervalos QT e QTc foram excluídos do estudo.

### Análise Estatística

A análise estatística foi realizada com os programas *Statistical Package for the Social Sciences* (SPSS) para Windows 20 (IBM SPSS Inc., Chicago, Illinois) e Medcalc 11.4.2 (MedCalc Software, Mariakerke, Bélgica). A conformidade dos dados com a distribuição normal foi testada com o teste de Kolmogorov-Smirnov. As variáveis numéricas normalmente distribuídas foram expressas como média ± desvio padrão. Variáveis categóricas foram expressas em números e porcentagens. Para as comparações entre o grupo usuário de heroína e os grupos de controle, o teste-t não pareado foi usado. Os testes de qui-quadrado e exato de Fisher foram realizados para comparar variáveis categóricas. Para as comparações de ambos os períodos pré-tratamento e pós-tratamento, o teste de McNemar e amostras pareadas de teste t foram usados. Um valor de p <0,05 foi aceito como estatisticamente significativo.

## Resultados

A distribuição da população estudada (n=136, idade média 30,40±9,58) foi a seguinte: 66 (48,52%) Heroína (+) e 70 (51,47%) Heroína (-). O sexo feminino correspondeu a 8,82% da população estudada. Não houve diferença significativa na distribuição da média de idade entre os grupos. No grupo usuário de heroína, a duração média do uso da heroína foi de cinco anos. Estatisticamente, não há diferenças significativas entre os grupos em relação ao sexo, tabagismo, fração de ejeção do ventrículo esquerdo e doença cerebrovascular. Nenhuma diferença foi determinada nas outras características demográficas e laboratoriais entre os dois grupos ( Tabela 1 ).

**Tabela 1:** – Groups’ Baseline Characteristics and Laboratory Findings

Variáveis	Heroína (+) (n= 66)	Heroína (-) (n= 70)	valor de p
**Características base**			
Idade (anos), média (DP)	30,2±10,1	30,6±9,1	0,808
Sexo (feminino), n (%)	3(4,5%)	9(12,8%)	0,087
Fumantes atuais, n (%)	35(53,0%)	31(44,2%)	0,307
Doença arterial coronária, n (%)	0	0	-
Hipertensão, n (%)	0	0	-
Diabetes Mellitus, n (%)	0	0	-
Doença cerebrovascular, n (%)	1(1,5%)	2(2,8%)	0,594
Fração de ejeção do ventrículo esquerdo (%)	59,8±2,9	60,4±9,4	0,620
**Resultados laboratoriais**			
Sódio (mmol/dl; DP)	139,48±4,81	140,37±5,20	0,302
Potássio (mmol/dl; DP)	4,32±0,51	4,45±0,65	0,198
Cálcio (mg/dl; DP)	9,45±0,82	9,53±0,93	0,596
Magnésio (mg/dl; DP)	1,99±0,30	2,02±0,26	0,533
Creatinina (mg/dl; DP)	0,77±0,22	0,72±0,23	0,197
HDL-C (mg/dl; DP)	37,61±8,45	38,89±10,53	0,437
LDL-C (mg/dl; DP)	137,74±39,81	125,66±45,14	0,101
Triglicerídeo (mg/dl; DP)	155,42±96,50	163,44±85,55	0,608
GB (x10^3^ /µL; DP)	6,89±4,03	7,12±4,35	0,750
Hemoglobina (g/dL; DP)	14,31±4,38	14,53±2,74	0,724
Hematócrito, n (%;DP)	42,53±4,11	42,88±5,43	0,673
Plaquetas (x10^3^ /µL; DP)	253,75±68,32	261,16±77,14	0,555
LDH %	15,23±2,06	13,88±1,78	0,001
TSH (uIU/mL)	2,12±1,77	2,16±1,98	0,896

*Teste-t de amostras independentes, Teste do qui-quadrado, Teste exato de Fisher *p<0,05 estatisticamente significativo. As variáveis contínuas são relatadas como média ± DP). Variáveis categóricas são relatadas como n (%). HDL-C: Colesterol de lipoproteína de alta densidade; LDL-C: Colesterol de lipoproteína de baixa densidade; GB: Glóbulos brancos; LDH: Largura de distribuição de hemácias; TSH: Hormônio estimulador da tireóide.

A comparação dos achados eletrocardiográficos entre os grupos revelou que não houve diferença estatística entre os grupos em termos de período PR, alterações inespecíficas do segmento ST-onda T, duração do QRS e tempo de pico da onda R. No grupo usuário de heroína (+), os intervalos QT (341,50±25,80 *versus* 379,11±45,23, p<0,01) e QTc (385,12±29,11 versus 411,3±51,70, p<0,01) os intervalos foram significativamente mais curtos do que o grupo usuário de heroína (-). O tempo T-pico até o T-final foi significativamente menor no grupo heroína (+), em comparação com o grupo heroína (-) (65,41±10,82 versus 73,3±10,13, p<0,01). Nenhuma diferença significativa foi observada entre os dois grupos em termos de razões Tpe/QT e Tpe/QTc ( Tabela 2 ).

**Tabela 2:** – Achados de ECG dos grupos

Achados de ECG	Heroína (+) (n= 66)	Heroína (-) (n= 70)	valor de p
Frequência cardíaca, bat/min	77,25±12,84	71,43±11,22	0,02
PR, msc	147,83±29,49	151,12±33,19	0,54
Alterações não específicas da onda T do segmento ST, n (%)	8(12,1%)	4(5,7%)	0,18
QRS, msg	98,82±19,53	100,50±19,87	0,49
QT, msg	341,50±25,80	379,11±45,23	<0,01
QTc, msg	385,12±29,11	411,3±51,70	<0,01
Tpe, msg	65,41±10,82	73,3±10,13	<0,01
Horário de pico da onda R, msg	32,18±8,16	34,22±9,32	0,17
Tpe/QT	0.19±0.03	0.2±0.03	0.70
Tpe/QTc	0.17±0.03	0.18±0.03	0.15

*Teste-t de amostras independentes; Teste de qui-quadrado; Teste exato de Fisher; Variáveis contínuas são relatadas como média±DP). Variáveis categóricas são relatadas como n (%); p<0,05 estatisticamente significativo.

Um total de 16 pacientes completou o tratamento com sucesso. Na análise de subgrupo desse grupo, os intervalos QT (356,81±37,49 *versus* 381,18±40,03, p<0,01) e QTc (382,06±26,41 versus 396,06±29,80, p<0,01) foram significativamente mais curtos no período pré-tratamento. Nenhuma diferença foi determinada nos outros parâmetros eletrocardiográficos entre os períodos pré e pós-tratamento ( Tabela 3 ).

**Tabela 3:** – Achados de ECG dos pacientes tratados

Variáveis	Antes do tratamento (n= 16)	Depois do tratamento (n= 16)	valor de p
Frequência cardíaca, bat/min	79,06±9,08	74,81±8,37	0,02
PR, msc	148,75±18,65	150,01±19,15	0,13
Alterações não específicas da onda T do segmento ST, n (%)	3(18,7)	3(18,7)	1,00
QRS, msg	98,08±10,58	98,68±8,80	0,46
QT, msg	356,81±37,49	381,18±40,03	<0,01
QTc, msg	382,06±26,41	396,06±29,80	<0,01
Tpe, msg	64,75±8,10	66,37±9,68	0,28
Horário de pico da onda R, msg	33,06±4,80	33,93±5,10	0,42
Tpe/QT	0,18±0,02	0,17±0,02	0,18
Tpe/QTc	0,17±0,02	0,18±0,02	0,23

*Teste de McNemar, teste-t de amostras pareadas. Variáveis contínuas são relatadas como média±DP, e variáveis categóricas são relatadas como n (%); p<0,05 estatisticamente significativo.

## Discussão

Atualmente, o vício em heroína é um problema de saúde preocupante, e as informações sobre os efeitos eletrocardiográficos da heroína são limitadas. Até onde sabemos, o presente estudo é o primeiro na literatura sobre alterações eletrocardiográficas dependentes de heroína. No estudo, mostramos que o vício em heroína afeta significativamente os intervalos de tempo QT, QTc e Tpe.

O vício em heroína é responsável por eventos cardíacos. O efeito do uso de heroína nas funções cardíacas foi investigado anteriormente em alguns estudos. O uso de heroína mostrou aumentar significativamente a taxa de anormalidades das válvulas mitral e tricúspide.^11^ Demirkıran et al.,^12^ demonstraram que os canabinóides sintéticos afetaram negativamente a função ventricular esquerda, ao passo que a heroína não.^12^ O uso de heroína não parece ter efeito sobre as funções ventriculares esquerdas de acordo com os resultados desses estudos; por outro lado, irregularidades atriais e miocárdicas com amostra histopatológica^13^ e contagem de leucócitos miocárdicos intersticiais, contagem de linfócitos T e macrófagos promovem aumento de cinco vezes nas amostras miocárdicas. ^14^ Os efeitos cardíacos da heroína não se limitam aos efeitos miotóxicos. Pavlidis et al.,^15^ relataram que infarto do miocárdio pode ser observado, embora raramente, para o qual o mecanismo comum é desconhecido.^15^ Orlando et al.,^16^ relataram redução subclínica da fração de ejeção do ventrículo esquerdo em 20 indivíduos dependentes de heroína.^16^ No entanto, esses estudos não fornecem informações sobre o efeito da dependência de heroína nos parâmetros eletrocardiográficos. Além disso, em estudos que investigaram os mecanismos de arritmias relacionadas à heroína e subsequentes mortes súbitas, o uso de heroína não só levou à infiltração miocárdica, mas também levou à displasia fibromuscular no nó sinusal, nó atrioventricular e vias de transmissão, e à infiltração de gordura. Os autores concluíram que essa pode ser a causa da arritmia relacionada à morte súbita em indivíduos dependentes de heroína.^9 , 10^ Portanto, revelar alterações no ECG dependentes de heroína tornou-se ainda mais importante.

Em um dos primeiros estudos sobre os efeitos eletrocardiográficos da dependência de heroína, Glauser et al.,^17^ mostraram que os achados mais comuns foram alterações inespecíficas de ST-T em 17 pacientes; e taquicardia sinusal em 11 pacientes.^17^ No entanto, os parâmetros eletrocardiográficos como QT, QTc, tempo de Tpe e durações de QSR não foram examinados neste estudo. Em um relato de caso, os autores mostraram que a overdose de heroína é uma possível causa da fenocopia de Brugada.^18^ Embora alguns estudos tenham sido realizados em ratos e cães, não há muitas informações na literatura sobre eletrocardiogramas em humanos. No presente estudo, o vício em heroína mostrou diminuir significativamente os intervalos de tempo QT, QTc e Tpe. A síndrome do QT curto, como o prolongamento do QT, também é bem conhecida por sua associação a arritmias cardíacas graves e morte súbita cardíaca.^19 - 21^ Além disso, o intervalo Tpe foi proposto como um marcador não invasivo de risco arrítmico. O intervalo do pico T ao final do T no eletrocardiograma (ECG) é uma medida da dispersão miocárdica da repolarização. Evidências crescentes sugerem que o intervalo Tpe pode predizer a suscetibilidade à arritmia em pacientes com várias doenças cardiovasculares.^22^ Marjamaa et al.,^23^ concluíram que o alelo menor da variante comum rs7219669 está associado ao encurtamento do intervalo Tpe em duas populações de estudo independentes, sendo, portanto, um candidato a modular a suscetibilidade à arritmia em nível populacional.^23^ No presente estudo, o uso de heroína teve um efeito significativo sobre esses parâmetros importantes. Usuários de heroína apresentam redução da modulação vagal cardíaca, e a terapia com metadona aumentou a atividade vagal diretamente em indivíduos que tiveram recidiva recente de uso de heroína.^8^ Além dos achados inflamatórios aumentados do miocárdio, também acreditamos que essa diminuição na atividade vagal possa ser responsável por alterações no ECG. A maioria dos estudos sobre dependência de heroína e ECG dizem respeito à metadona. A metadona é usada no tratamento da dependência de heroína, e um de seus efeitos conhecidos mais importantes é o prolongamento das durações QT e QTc.^24 , 25^ A metadona é um inibidor do canal iônico cardíaco KCNH226 e causa o prolongamento do intervalo QT de maneira relacionada à dose consumida.^26^ Por outro lado, pode aumentar a atividade vagal e prolongar a duração do intervalo QT.^8^ Após o presente estudo, acreditamos que parte dos efeitos da metadona pode estar relacionada à neutralização do efeito da heroína. Nosso estudo demonstrou que o vício em heroína mudou significativamente os intervalos QT, QTc e Tpe, independentemente do efeito dos adulterantes e da metadona. Além de indivíduos dependentes de heroína, a metadona também é conhecida por prolongar o intervalo QT. No entanto, no grupo de usuários de heroína, esse resultado deve ser levado em consideração ao discutir o efeito de prolongamento do intervalo QT da metadona em pessoas dependentes de heroína. Embora o efeito da heroína nos canais de potássio seja desconhecido, o efeito na atividade vagal foi analisado.^8^ Com esses resultados, embora ela não seja responsável por todos os mecanismos de prolongamento do intervalo QT, acreditamos que a neutralização dos efeitos da heroína contribui significativamente para o prolongamento do QT. Com o presente estudo, não podemos explicar exatamente se o prolongamento QT é efeito direto da metadona ou o resultado da neutralização do efeito da heroína em estudos anteriores. Quando consideramos o assunto um novo ponto de vista, é um tema importante para orientar os demais estudos. Ainda assim, a metadona é um agonista opioide completo, e as mortes por overdose são um grande problema. A buprenorfina, um agonista opioide parcial, tem se tornado uma opção cada vez mais popular na prática clínica em nosso país e em todo o mundo. A buprenorfina é, provavelmente, o agente mais seguro devido à sua ação farmacológica única e foi declarada uma nova opção para o tratamento da dependência de heroína com menor potencial de abuso e baixo risco de overdose.^27^ Doses terapêuticas de buprenorfina mostraram não ter efeitos sobre a duração de QT e QTc,^28 , 29^ e a buprenorfina em doses comumente utilizadas é uma alternativa adequada à metadona, no que diz respeito ao risco de prolongamento do intervalo QTc.^30^ Portanto, os indivíduos do presente estudo foram tratados com buprenorfina em vez de metadona. A buprenorfina não teve efeitos significativos nas durações de QT e QTc, o que foi uma das vantagens do presente estudo. Visto isso, observamos os efeitos da heroína no ECG com mais clareza. Quando olhamos para o subgrupo de tratamento, há uma mudança significativa nas durações de QT e QTc devido à interrupção do uso da heroína. Se esses indivíduos fossem tratados com metadona, seria muito difícil entender se esse efeito era devido à heroína ou à metadona. Infelizmente, assim como em outros países, a desvantagem mais importante nesse subgrupo é a proporção de pacientes que podem concluir o tratamento médico para dependência de heroína, insuficiente em nosso estudo.^31^ A maioria dos pacientes não conseguiu completar o tratamento, e é por isso que o número de indivíduos no subgrupo do estudo diminuiu significativamente. Embora tenha havido uma mudança significativa nos intervalos QT e QTc no grupo pós-tratamento, houve apenas um aumento numérico na duração do Tpe, o qual não atingiu significância estatística. Presumimos que isso seja devido ao número insuficiente de indivíduos que concluíram o tratamento. Porém, estudos com maior número de participantes são necessários para uma decisão definitiva sobre o assunto.

A heroína tem uma meia-vida extremamente curta no sangue (menos de cinco minutos) e é imediatamente convertida no metabólito ativo 6-acetilmorfina (6-AM), que é posteriormente metabolizado em morfina.^32^ Na urina, o metabólito ativo 6-AM pode ser detectado por um período mais longo, possivelmente até 12 horas.^33^ Portanto, foram incluídos no presente estudo pacientes que usaram heroína apenas nas últimas 12 horas. Por outro lado, quando a heroína é usada por via intravenosa, é administrada em conjunto com substâncias químicas adicionais, denominadas adulterantes (paracetamol, cafeína, difenidramina, metorfano, alprazolam, quetiapina, cloroquina, diltiazem, cocaína, procaína, lidocaína, quinina/quinidina, fenacetina e tiamina), e os potenciais efeitos cardíacos dessas substâncias complicam a avaliação dos efeitos eletrocardiográficos da heroína.^34^ Desa forma, para investigar os efeitos cardíacos da heroína apenas, excluímos aqueles que usaram heroína por injeção intravenosa. O presente estudo demonstrou que o uso de heroína diminuiu significativamente os intervalos QT, QTc e Tpe independentemente do efeito dos adulterantes.

### Limitações do Estudo

As principais limitações do presente estudo foram o desenho unicêntrico e o número relativamente menor de indivíduos.

## Conclusão

O vício em heroína afeta significativamente os intervalos de tempo QT, QTc e Tpe. Os efeitos de arritmia desses parâmetros já são conhecidos. Os parâmetros eletrocardiográficos desses indivíduos merecem mais atenção. Tendo em vista que o conhecimento atual sobre os efeitos do uso de heroína nas funções cardíacas é limitado, o estudo do assunto é imprescindível para sua contribuição para a literatura. Ainda assim, estudos futuros com uma amostra maior são necessários para alcançar consenso e resultados concretos.
