# Supplementary material for: Efeitos da Heroína nos Parâmetros Eletrocardiográficos
Source: Arq Bras Cardiol. 2020 Dec 1;115(6):1135–41. [Article in Portuguese] doi: 10.36660/abc.20190296 (PMC8133719; doi:10.36660/abc.20190296)
Supplement: Translation

# Translation: Effect of Heroin on Electrocardiographic Parameters

**Authors:** Ersin Yildirim, Murat Selcuk, Faysal Saylik, Ferit Onur Mutluer, Ozgur Deniz

**Affiliations:** 1 Turkey Umraniye Training and Research Hospital, Istanbul - Turkey; 2 Turkey Van Egitim ve Arastirma Hastanesi, Van – Turkey

**Keywords:** Heroin, Heroin Dependence, Narcotics/toxicity, Long Qt Syndrome, Arrhythmias, Cardiac/adverse effects, Pulmonary Edema, Renal Insufficiency, Leukoencephalopaties

## Abstract

**Background:**

Heroin addiction is currently a significant health problem, and information on the electrocardiographic effects of heroin is limited.

**Objetivo:**

The aim of the present study is to investigate effects of heroin addiction on electrocardiographic parameters.

**Methods:**

A total of 136 individuals, including 66 individuals who smoke heroin as the study group and 70 healthy individuals with no drug addiction as the control group, were included in the study. Individuals who inject heroin were excluded. Electrocardiographic (ECG) evaluation of those using heroin was performed and compared with those of the control group. In addition, pre-treatment and post-treatment ECG of the heroin group were compared. A p-value of <0.05 was accepted as statistically significant.

**Results:**

Heart rate (77.2±12.8 *versus* 71.4±11.2; p=0.02) were found to be higher in the heroin group compared to the control group. QT (341.50±25.80 *versus* 379.11±45.23; p=0.01), QTc intervals (385.12±29.11 *versus* 411.3±51.70; p<0.01), and T peak to end time (Tpe) (65.41±10.82 *versus* 73.3±10.13; p<0.01) were significantly shorter in the heroin group. No difference was observed between the groups with regard to Tpe/QT and Tpe/QTc ratios. In the subgroup analysis of the heroin group, QT (356.81±37.49 *versus* 381.18±40.03; p<0.01) and QTc (382.06±26.41 *versus* 396.06±29.80; p<0.01) intervals were significantly shorter in the pre-treatment period.

**Conclusion:**

Heroin addiction significantly affects the QT, QTc, and Tpe time intervals. The arrhythmia effects of these parameters are well known. More attention to the electrocardiographic parameters of these individuals should be given. (Arq Bras Cardiol. 2020; 115(6):1135-1141)

## Introduction

Heroin, which is a central nervous system depressant (diacetylmorphine), is a semi-synthetic opiate. Heroin is a highly abused opioid, and heroin addiction incurs a significant detriment to society worldwide. The prevalence of heroin use has increased in recent years. Mortality among heroin users varies between 1 and 3%, and the most effective treatment method of heroin addiction is opioid replacement therapy.^1 , 2^Its main adverse effect is respiratory distress, which can lead to death. With loss of tolerance, heroin overdose can be lethal after a period of abstinence. The other various complications of heroin addiction, such as pulmonary edema,^3^shock,^4^myocardial damage, acute renal failure,^5^rhabdomyolysis,^6^and leukoencephalopathy^7^have been described in the literature. Besides that, heroin has proved to be effective on vagal modulation and autonomic regulation.^8^However, our knowledge of the cardiac effects of heroin addiction is limited, which is an important public health problem of this extent. There are also some studies in the literature showing the relation between heroin, myocardial toxicity, and arrhythmias.^9 , 10^Therefore, understanding heroin dependent ECG changes is essential. The aim of the present study is to investigate effects of heroin addiction on electrocardiographic parameters.

## Methods

After approval by the Ethics Committee, a total of 136 individuals were included in the study, which included 66 patients who use heroin via smoking and undergoing therapy in the Alcohol and Drug Addiction Treatment and Training Center, between 2014 and 2017, as the study group; and 70 healthy individuals with no drug addiction other than smoking as the control group. Control group was selected consecutively from the patients visiting the cardiology clinic. ECG evaluation of those using heroin was performed and compared to those of the control group. In addition, pre-treatment and post-treatment ECG of the heroin group was evaluated. The clinical and demographic characteristics of patients, status and duration of heroin addiction were collected from patients and their files in the hospital. Only those with heroin use via smoking were included in the study. Electrocardiography (ECG) records of patients were obtained with Schiller Cardiovit AT-102 plus, using the standard 12 derivation (10 mm/mV calibration and 25 mm/s sliding rate) at first admission to the hospital. Only the patients on heroin which was taken within 12 hours of the ECG records obtained were included the study. ECG measurements of QT and Tpe intervals were performed manually by two expert cardiologists, using a magnifying glass to decrease measurement errors. Leads 2 and V5 were selected for measuring QT and Tpe intervals, respectively. The average of the three beats in each ECG leads was calculated. QT interval is calculated as the interval from the beginning of the QRS to the end of the T wave. Tpe interval is defined as the interval from the peak of T wave to the end of T wave. QTc intervals were calculated using the Bazett formula. Complete blood count (CBC) and biochemical tests were performed using a Beckman Coulter LH-750 and a Beckman Coulter L × 20, respectively; the results of each patient were recorded. Echocardiographic evaluations of all patients were made at the first admission to the hospital. All participants underwent 2D and Doppler echocardiographic evaluation (VIVID 3, General Electric, USA) and the left ventricular ejection fraction was calculated using modified Simpson rules. Those who used heroin via the intravenous route, alcohol-dependent, those with coronary artery diseases, cardiac failure, cardiac valve disorders, known arrhythmias, hypertension, congenital cardiac diseases, diabetes, hepatic or renal failure, chronic obstructive pulmonary disease, endocrine diseases, metabolic or electrolyte disorders, acute or chronic infections, or patients that took medications which can affect QT and QTc intervals were excluded from the study.

### Statistical Analysis

Statistical analysis was performed with Statistical Package for Social Sciences (SPSS) for Windows 20 (IBM SPSS Inc., Chicago, IL) and Medcalc 11.4.2 (MedCalc Software, Mariakerke, Belgium) programs. Data compliance with the normal distribution was tested using the Kolmogorov-Smirnov test. The normally distributed numeric variables were expressed as mean ± standard deviation. Categorical variables were expressed as numbers and percentages. For the comparisons between the heroin and the control groups, unpaired student’s t test was used. Chi-square and Fisher’s exact tests were carried out to compare categorical variables. For the comparisons of both the pre-treatment and post-treatment periods, McNemar’s test and t-test paired samples were used. A *p* -value of <0.05 was accepted as statistically significant.

## Results

The distribution of the study population (n=136, mean age 30.40±9.58) was as following: 66 (48.52%) Heroin (+) and 70 (51.47%) Heroin (-). Females corresponded to 8.82% of the study population. There was no significant difference in the distribution of mean age between groups. In the heroin group, the mean duration of heroin use was five years. Statistically, there are not significant differences between the groups, related to the gender, smoking, left ventricular ejection fraction, and cerebrovascular disease. No difference was determined in the other demographic and laboratory characteristics between both groups ( Table 1 ).

**Table 1:** – Groups’ Baseline Characteristics and Laboratory Findings

Variables	Heroin (+) (n= 66)	Heroin (-) (n= 70)	p-value
**Baseline characteristics**			
Age (years old), mean (SD)	30.2±10.1	30.6±9.1	0.808
Gender (female), n (%)	3(4.5%)	9(12.8%)	0.087
Current Smoker, n (%)	35(53.0%)	31(44.2%)	0.307
Coronary Artery disease, n (%)	0	0	-
Hypertension, n (%)	0	0	-
Diabetes Mellitus, n (%)	0	0	-
Cerebrovascular Disease, n (%)	1(1.5%)	2(2.8%)	0.594
Left ventricular ejection fraction (%)	59.8±2.9	60.4±9.4	0.620
**Laboratory Findings**			
Sodium (mmol/dl; SD)	139.48±4.81	140.37±5.20	0.302
Potassium (mmol/dl; SD)	4.32±0.51	4.45±0.65	0.198
Calcium (mg/dl; SD)	9.45±0.82	9.53±0.93	0.596
Magnesium (mg/dl; SD)	1.99±0.30	2.02±0.26	0.533
Creatinine (mg/dl; SD)	0.77±0.22	0.72±0.23	0.197
HDL-C (mg/dl; SD)	37.61±8.45	38.89±10.53	0.437
LDL-C (mg/dl; SD)	137.74±39.81	125.66±45.14	0.101
Triglyceride (mg/dl; SD)	155.42±96.50	163.44±85.55	0.608
WBC (x10^3^/µL; SD)	6.89±4.03	7.12±4.35	0.750
Hemoglobin (g/dL; SD)	14.31±4.38	14.53±2.74	0.724
Hematocrit, n (%;SD)	42.53±4.11	42.88±5.43	0.673
Platelets (x10^3^/µL; SD)	253.75±68.32	261.16±77.14	0.555
RDW%	15.23±2.06	13.88±1.78	0.001
TSH(uIU/mL)	2.12±1.77	2.16±1.98	0.896

* Independent Samples T-Test, chi-square Test, Fisher’s Exact Test *p<0.05 statistically significant. Continues variables are reported mean ± SD). Categorical variables are reported n (%). HDL-C: High Density Lipoprotein Cholesterol; LDL-C: Low Density Lipoprotein Cholesterol; WBC: White Blood Cell; RDW: Red Cell Distribution Width; TSH: Thyroid Stimulating Hormone.

Comparison of the electrocardiographic findings between the groups revealed that there was no statistically difference between the groups in terms of PR period, nonspecific ST segment-T wave changes, QRS and R wave peak time durations. In heroin (+) group, QT (341.50±25.80 *versus* 379.11±45.23, p<0.01) and QTc (385.12±29.11 *versus* 411.3±51.70, p<0.01) intervals were significantly shorter than heroin (-) group. T-peak to T-end time was significantly shorter in the heroin (+) group, compared to the heroin (-) group (65.41±10.82 *versus* 73.3±10.13, p<0.01). No significant difference was observed between both groups in terms of Tpe/QT and Tpe/QTc ratios ( Table 2 ).

**Table 2:** – ECG Findings of the Groups

ECG Findings	Heroin (+) (n= 66)	Heroin (-) (n= 70)	p-value
Heart Rate, beat/min	77.25±12.84	71.43±11.22	0.02
PR, msc	147.83±29.49	151.12±33.19	0.54
Nonspecific ST segment-T wave changes, n (%)	8(12.1%)	4(5.7%)	0.18
QRS, msc	98.82±19.53	100.50±19.87	0.49
QT, msc	341.50±25.80	379.11±45.23	<0.01
QTc, msc	385.12±29.11	411.3±51.70	<0.01
Tpe, msc	65.41±10.82	73.3±10.13	<0.01
R wave peak time, msc	32.18±8.16	34.22±9.32	0.17
Tpe/QT	0.19±0.03	0.2±0.03	0.70
Tpe/QTc	0.17±0.03	0.18±0.03	0.15

*Independent Samples T-Test, Chi-square Test, Fisher’s Exact Test; Continues variables are reported as mean ± SD). Categorical variables are reported as n (%); p<0.05 statistically significant.

A total of 16 patients completed the treatment successfully. In the subgroup analysis of this group, QT (356.81±37.49 *versus* 381.18±40.03, p<0.01) and QTc (382.06±26.41 *versus* 396.06±29.80, p<0.01) intervals were significantly shorter in the pre-treatment period. No difference was determined in the other electrocardiographic parameters between both the pre-treatment and posttreatment periods ( Table 3 ).

**Table 3:** – ECG findings of treated patients

Variables	Before Treatment (n= 16)	After Treatment (n= 16)	p-value
Heart Rate, beat/min	79.06±9.08	74.81±8.37	0.02
PR, msc	148.75±18.65	150.01±19.15	0.13
Nonspecific ST segment-T wave changes, n (%)	3(18.7)	3(18.7)	1.00
QRS, msc	98.08±10.58	98.68±8.80	0.46
QT, msc	356.81±37.49	381.18±40.03	<0.01
QTc, msc	382.06±26.41	396.06±29.80	<0.01
Tpe, msc	64.75±8.10	66.37±9.68	0.28
R wave peak time, msc	33.06±4.80	33.93±5.10	0.42
Tpe/QT	0.18±0.02	0.17±0.02	0.18
Tpe/QTc	0.17±0.02	0.18±0.02	0.23

* McNemar’s test, paired samples t-test. Continues variables are reported as mean ± SD, and categorical variables are reported as n (%); p<0.05 statistically significant.

## Discussion

Today, heroin addiction is a significant health problem. However, despite that, information on the electrocardiographic effects of heroin is limited. To the best of our knowledge, the present study is the first in literature about heroin-dependent electrocardiographic changes. In this study, we showed that heroin addiction significantly affects QT, QTc, and Tpe time intervals.

Heroin addiction is responsible for cardiac events. The effect of heroin use on cardiac functions has been previously investigated in some studies. Heroin use was shown to significantly increase the rate of mitral and tricuspid valve abnormalities.^11^Demirkıran et al.^12^demonstrated that synthetic cannabinoids negatively affected the left ventricular function, whereas heroin did not.^12^Heroin use does not seem to have any effects on the left ventricular functions according to the results of these studies; on the other hand, atrial and myocardial irregularities with histopathological sampling^13^and interstitial myocardial leukocyte count, T lymphocyte and macrophage counts were observed to promote a five-fold increase in myocardial samples.^14^The cardiac effects of heroin are not limited to myotoxic effects. Pavlidis et al.^15^reported that myocardial infarction could be observed, even though rarely, in which the common mechanism is unknown.^15^Orlando et al.^16^reported a subclinical reduction in the ejection fraction of the left ventricle in 20 heroin-dependent individuals.^16^However, these studies do not provide any information about the effect of heroin addiction on electrocardiographic parameters. Furthermore, in studies investigating the mechanisms of heroin-related arrhythmias and subsequent sudden deaths, heroin use did not only lead to myocardial infiltration, but it also led to fibromuscular dysplasia in the sinus node, atrioventricular node and transmission pathways, and to fat infiltration; they concluded that this may be the cause of arrhythmia related to sudden death in heroin-dependent individuals.^9 , 10^Therefore, revealing heroin-dependent ECG changes has become even more important.

In one of the first studies on the electrocardiographic effects of heroin dependence, Glauser et al.^17^showed that the most common findings were nonspecific ST-T changes in 17 patients; sinus tachycardia, in 11 patients.^17^However, electrocardiographic parameters such as QT, QTc, Tpe time, and QSR durations have not been examined in this study. In a case report, authors showed that heroin overdose is a possible cause of Brugada phenocopy.^18^Although some studies have been performed on mice and dogs, there is not much literature information about electrocardiograms in humans. In the present study, heroin addiction was shown to significantly decrease QT, QTc, and Tpe time intervals. The short QT syndrome, such as QT prolongation, is also well known for its association to severe cardiac arrhythmias and sudden cardiac death.^19 - 21^In addition, the Tpe interval has been proposed as a non-invasive marker of arrhythmic risk. T-peak to T-end interval on the electrocardiogram (ECG) is a measure of myocardial dispersion of repolarization. Increasing evidence suggests that Tpe interval may predict arrhythmia susceptibility in patients with various cardiovascular diseases.^22^Marjamaa et al.^23^concluded that the minor allele of common variant rs7219669 is associated to Tpe interval shortening in two independent study populations, thus being a candidate to modulate arrhythmia susceptibility at population level.^23^In the present study, heroin use was found to have a significant effect on these important parameters. Heroin users show reduced cardiac vagal modulation, and methadone therapy raised vagal activity directly in individuals who had recently relapsed into heroin use.^8^In addition to the increased myocardium inflammatory findings, we also believe that this decrease in vagal activity may be responsible for ECG changes. Most studies on heroin addiction and ECG are about methadone. Methadone is used in the treatment of heroin addiction, and one of its most important known effects is the prolongation of QT and QTc durations.^24 , 25^Methadone is an inhibitor of the cardiac ion channel KCNH226 and causes QT prolongation in a dose-dependent manner.^26^On the other hand, it can increase vagal activity and prolong QT duration.^8^After the present study, we believe that a part of methadone’s effects may be related to the neutralization of the heroin effect. Our study demonstrated that heroin addiction significantly changed QT, QTc, and Tpe intervals independent from the effect of adulterants and methadone. In addition to heroin dependent individuals, methadone is also known to prolong QT. However, in heroin user group, this result should be taken into consideration when discussing the QT prolonging effect of methadone in those who depend on heroin. Although the effect of heroin on potassium channels is unknown, the effect on vagal activity has been shown.^8^With these results, even though it is not responsible for all QT prolongation mechanism, we think that neutralizing heroin effects significantly contributes to QT prolongation. With the present study, we cannot explain exactly whether QT prolongation is the direct effect of methadone or the result of the neutralization of the heroin effect in previous studies. In terms of bringing a new point of view, it is an important subject in guiding the other studies. On the other hand, methadone is a complete opioid agonist, and overdose deaths are a major problem. Buprenorphine, a partial opioid agonist, has become an increasingly popular option in clinical practice in our country and all over the world. Buprenorphine is probably the safer agent due to its unique pharmacological action and has been declared a new dawn for treating heroin dependence with less abuse potential and low overdose risk.^27^Therapeutic doses of buprenorphine were shown to have no effects on QT and QTc duration,^28 , 29^and buprenorphine in commonly used doses is a suitable alternative to methadone, with regard to the risk of QTc prolongation.^30^Therefore, individuals from the present study were treated with buprenorphine instead of methadone. Buprenorphine had no significant effects on QT and QTc durations, which was one of our study advantages. Seen that, we observed the effects of heroin on ECG more clearly. When we look at the treatment subgroup, there is a significant change in QT and QTc durations because of heroin discontinuation. If these individuals were treated with methadone, understanding whether this effect was due to heroin or methadone would be very difficult. Unfortunately, like in other countries, the most important disadvantage in this subgroup is the proportion of patients who can complete medical heroin-addiction treatment, which is insufficient in our study.^31^Most patients could not complete the treatment, and that is why the number of individuals in the study subgroup decreased significantly. Although there was a significant change in QT and QTc in the post-treatment group, there was only a numerical increase in Tpe duration, and this increase did not reach statistical significance. We assume this is due to insufficient number of individuals who have completed the treatment. However, studies with a larger number of participants are needed to make a definite decision on this issue.

Heroin has an extremely short half-life in blood (less than five minutes), and is immediately converted to the active metabolite 6-acetylmorphine (6-AM), which is further metabolized to morphine.^32^In urine, active metabolite 6-AM can be detected for a longer period, possibly up to 12 hours.^33^Therefore, patients who had used heroin only in the last 12 hours were included in the present study. On the other hand, when heroin is used via the intravenous route, it is administered together with additional chemical substances, named as adulterants (acetaminophen, caffeine, diphenhydramine, methorphan, alprazolam, quetiapine, chloroquine, diltiazem, cocaine, procaine, lidocaine, quinine/quinidine, phenacetine, and thiamine), and the potential cardiac effects of these substances complicate the evaluation of heroin electrocardiographic effects.^34^Thus, in order to investigate the cardiac effects of only heroin, we excluded those who used heroin by intravenous injection. The present study demonstrated that heroin use significantly decreased the QT, QTc and Tpe intervals independent from the effect of adulterants.

### Study limitations

Our study had some limitations: the single-center design and the relatively lower number of individuals are the most important ones.

## Conclusion

Heroin use is a serious public health issue, which significantly affects the QT, QTc, and Tpe time intervals. The arrhythmia effects of these parameters are well known, and we should be more alert to the electrocardiographic parameters of these individuals. Given that the present knowledge on the effects of heroin use on cardiac functions is limited, studying the matter is imperative for its contribution to the literature. Nonetheless, further studies with larger sample sizes are needed for a consensus and clear results.
